# Supplementary material for: Gall Apparency Drives Parasitoid Richness in a Highly Specialised Gall‐Parasitoid Food Web From a Tropical Rainforest
Source: Ecol Evol. 2026 Oct 6;16(10):e74375. doi: 10.1002/ece3.74375 (PMC13640053; doi:10.1002/ece3.74375)
Supplement: Supplementary file 1 [file ECE3-16-e74375-s001.zip › ece-2026-03-00658-manifest.html]

Manifest file for export ece-2026-03-00658-20260911081540


|  |  |
| --- | --- |
| File name: ece-2026-03-00658-20260911081540 | |
| Export Date: 11-Sep-2026 | |
| Output Format: XML (ScholarOne DTD) | |
| ece-2026-03-00658-20260911081540/doc/01\_MS\_revised\_CLEAN.docx | Version 1.0 |
| ece-2026-03-00658-20260911081540/suppl\_data/04\_MS\_supplementary.docx | Version 1.0 |
| ece-2026-03-00658-20260911081540/suppl\_data/03\_MS\_response\_to\_reviewers.docx | Version 1.0 |
| ece-2026-03-00658-20260911081540/suppl\_data/README.md | Version 1.0 |
| ece-2026-03-00658-20260911081540/suppl\_data/spp-accumulation-curve.R | Version 1.0 |
| ece-2026-03-00658-20260911081540/suppl\_data/requirements.txt | Version 1.0 |
| ece-2026-03-00658-20260911081540/suppl\_data/gall-para-interactions.csv | Version 1.0 |
| ece-2026-03-00658-20260911081540/suppl\_data/gall-traits.csv | Version 1.0 |
| ece-2026-03-00658-20260911081540/suppl\_data/host-gall-interactions.csv | Version 1.0 |
| ece-2026-03-00658-20260911081540/suppl\_data/inc-freq-by-sp.csv | Version 1.0 |
| ece-2026-03-00658-20260911081540/suppl\_data/compartments\_gall\_para.png | Version 1.0 |
| ece-2026-03-00658-20260911081540/suppl\_data/DHARMa\_cecidomyiidae\_only.png | Version 1.0 |
| ece-2026-03-00658-20260911081540/suppl\_data/DHARMa\_final\_apparency.png | Version 1.0 |
| ece-2026-03-00658-20260911081540/suppl\_data/DHARMa\_final\_size\_sensitivity.png | Version 1.0 |
| ece-2026-03-00658-20260911081540/suppl\_data/glm\_residuals\_final\_app.png | Version 1.0 |
| ece-2026-03-00658-20260911081540/suppl\_data/tripartite\_compartments\_S2.png | Version 1.0 |
| ece-2026-03-00658-20260911081540/suppl\_data/tripartite\_compartment\_membership.csv | Version 1.0 |
| ece-2026-03-00658-20260911081540/suppl\_data/dprime\_log.txt | Version 1.0 |
| ece-2026-03-00658-20260911081540/suppl\_data/dprime\_wilcoxon.txt | Version 1.0 |
| ece-2026-03-00658-20260911081540/suppl\_data/gall-traits-revision\_log.txt | Version 1.0 |
| ece-2026-03-00658-20260911081540/suppl\_data/model\_coefficients.csv | Version 1.0 |
| ece-2026-03-00658-20260911081540/suppl\_data/model\_comparison\_table.csv | Version 1.0 |
| ece-2026-03-00658-20260911081540/suppl\_data/model\_summaries.txt | Version 1.0 |
| ece-2026-03-00658-20260911081540/suppl\_data/parasitoid\_d\_prime.csv | Version 1.0 |
| ece-2026-03-00658-20260911081540/suppl\_data/compartments\_overview.py | Version 1.0 |
| ece-2026-03-00658-20260911081540/suppl\_data/pt\_nxlab.py | Version 1.0 |
| ece-2026-03-00658-20260911081540/suppl\_data/pypartite.py | Version 1.0 |
| ece-2026-03-00658-20260911081540/suppl\_data/tripartite-web.py | Version 1.0 |
| ece-2026-03-00658-20260911081540/suppl\_data/tripartite\_compartments\_S2.py | Version 1.0 |
| ece-2026-03-00658-20260911081540/suppl\_data/\_\_init\_\_.py | Version 1.0 |
| ece-2026-03-00658-20260911081540/suppl\_data/check-compartments.R | Version 1.0 |
| ece-2026-03-00658-20260911081540/suppl\_data/gall-para-web-PAC.R | Version 1.0 |
| ece-2026-03-00658-20260911081540/suppl\_data/gall-traits-full-model.r | Version 1.0 |
| ece-2026-03-00658-20260911081540/suppl\_data/gall-traits-revision.R | Version 1.0 |
| ece-2026-03-00658-20260911081540/suppl\_data/gall-traits-taxon-apparency-stackplot.R | Version 1.0 |
| ece-2026-03-00658-20260911081540/suppl\_data/specialisation-dprime-test.R | Version 1.0 |
| ece-2026-03-00658-20260911081540/pdf/ece-2026-03-00658.pdf |  |
| ece-2026-03-00658-20260911081540/ece-2026-03-00658-metadata.xml |  |
| ece-2026-03-00658-20260911081540/s1.dtd |  |
| manifest.html | This document |
