# Supplementary material for: Gall Apparency Drives Parasitoid Richness in a Highly Specialised Gall‐Parasitoid Food Web From a Tropical Rainforest
Source: Ecol Evol. 2026 Oct 6;16(10):e74375. doi: 10.1002/ece3.74375 (PMC13640053; doi:10.1002/ece3.74375)
Supplement: Supplementary file 1 [file ECE3-16-e74375-s001.zip › ece374375-sup-0001-TableS1-S4-FigureS1-S2@04_MS_supplementary.docx]

**Supplementary Information**

## Table S1.

Taxonomic information for 32 plant species sampled, along with their associated gall-forming insect herbivores, listed in host plant phylogenetic order from basal to derived species (as per in Fig. 1). Gall trait categories are listed in columns *taxon*, *module*, *pubescence*, *apparency* & *open*.

| No. | Host code | Clade | Plant family | Plant species | Gall code | Gall higher taxon | Gall family | Gall species | Taxon^3^ | Module^4^ | Pubescence^5^ | Apparency^6^ | Open^7^ |
| --- | --- | --- | --- | --- | --- | --- | --- | --- | --- | --- | --- | --- | --- |
| 1 | GNE | Basal | Gnetaceae | *Gnetum gnemon* | GALL039 | Diptera | Cecidomyiidae |  | CECI | LEAF | NOT | HI | F |
|  |  |  |  |  | GALL040 | Diptera | Cecidomyiidae |  | CECI | LEAF | NOT | LO | F |
| 2 | STG | Basal | Monimiaceae | *Kibara cf. coriacea* | GALL122 | Diptera | Cecidomyiidae |  | CECI | LEAF | NOT | LO | F |
| 3 | MYL | Basal | Myristicaceae | *Paramyristica cf. sepicana* | GALL077 | Diptera | Cecidomyiidae |  | CECI | LEAF | NOT | MID | F |
|  |  |  |  |  | GALL078 | Diptera | Cecidomyiidae |  | CECI | OTHER | NOT | HI | F |
|  |  |  |  |  | GALL079 | Diptera | Cecidomyiidae |  | CECI | COMP | NOT | MID | F |
|  |  |  |  |  | GALL080 | Insecta |  |  | UNKN | LEAF | NOT | LO | F |
|  |  |  |  |  | GALL081 | Hemiptera |  |  | HEMI | LEAF | NOT | LO | T |
|  |  |  |  |  | GALL173 | Thysanoptera |  |  | OTHE | LEAF | NOT | MID | T |
| 4 | DRA | Basal | Asparagaceae | *Dracaena angustifolia* | GALL038 | Diptera | Cecidomyiidae |  | CECI | LEAF | NOT | LO | F |
| 5 | COR | Basal | Asparagaceae | *Cordyline fruticosa* | GALL031 | Hemiptera |  |  | HEMI | COMP | NOT | MID | T |
| 6 | ARE | Basal | Arecaceae | *Hydriastele wendlandiana* | GALL161 | Insecta |  |  | UNKN | LEAF | NOT | LO | T |
| 7 | HOR | Basal | Zingiberaceae | *Hornstedtia scottiana* |  |  |  |  |  |  |  |  |  |
| 8 | MAA | Rosids I | Euphorbiaceae | *Macaranga aleuritoides* | GALL058 | Diptera | Cecidomyiidae |  | CECI | LEAF | NOT | HI | F |
|  |  |  |  |  | GALL059 | Diptera | Cecidomyiidae | *Schizomyia novoguinensis* Kolesik**^1^** | CECI | OTHER | NOT | MID | F |
|  |  |  |  |  | GALL062 | Diptera | Cecidomyiidae |  | CECI | LEAF | HAIRY | MID | F |
|  |  |  |  |  | GALL063 | Diptera | Cecidomyiidae |  | CECI | LEAF | HAIRY | HI | F |
| 9 | MAQ | Rosids I | Euphorbiaceae | *Macaranga quadriglandulosa* | GALL069 | Diptera | Cecidomyiidae |  | CECI | LEAF | NOT | LO | F |
|  |  |  |  |  | GALL070 | Diptera | Cecidomyiidae |  | CECI | OTHER | NOT | MID | F |
| 10 | MAU | Rosids I | Euphorbiaceae | *Macaranga novoguineensis* | GALL072 | Diptera | Cecidomyiidae |  | CECI | LEAF | HAIRY | MID | T |
|  |  |  |  |  | GALL075 | Insecta |  |  | UNKN | LEAF | HAIRY | MID | F |
|  |  |  |  |  | GALL170 | Insecta |  |  | UNKN | LEAF | HAIRY | LO | F |
|  |  |  |  |  | GALL171 | Diptera | Cecidomyiidae |  | CECI | LEAF | NOT | LO | F |
|  |  |  |  |  | GALL215 | Diptera | Cecidomyiidae |  | CECI | LEAF | HAIRY | MID | F |
|  |  |  |  |  | GALL216 | Diptera | Cecidomyiidae |  | CECI | LEAF | HAIRY | HI | F |
| 11 | MAL | Rosids I | Euphorbiaceae | *Mallotus mollissimus* | GALL064 | Diptera | Cecidomyiidae |  | CECI | LEAF | HAIRY | HI | F |
| 12 | HON | Rosids I | Euphorbiaceae | *Homalanthus novoguineensis* | GALL166 | Insecta |  |  | UNKN | LEAF | NOT | LO | T |
| 13 | PTE | Rosids I | Fabaceae | *Pterocarpus indicus* | GALL008 | Diptera | Cecidomyiidae |  | CECI | OTHER | NOT | LO | F |
|  |  |  |  |  | GALL009 | Diptera | Cecidomyiidae |  | CECI | COMP | NOT | MID | F |
| 14 | ART | Rosids I | Moraceae | *Artocarpus altilis* | GALL187 | Insecta |  |  | UNKN | LEAF | HAIRY | LO | F |
| 15 | PUN | Rosids I | Moraceae | *Ficus pungens* | GALL002 | Hemiptera |  |  | HEMI | COMP | HAIRY | LO | T |
| 16 | VAR | Rosids I | Moraceae | *Ficus variegata* | GALL003 | Hemiptera | Triozidae | *Pauropsylla udei* Rübsaamen | HEMI | LEAF | NOT | HI | F |
|  |  |  |  |  | GALL004 | Diptera | Cecidomyiidae |  | CECI | LEAF | NOT | MID | F |
|  |  |  |  |  | GALL005 | Hemiptera |  |  | HEMI | LEAF | NOT | LO | F |
|  |  |  |  |  | GALL006 | Hemiptera |  |  | HEMI | OTHER | NOT | LO | T |
| 17 | COP | Rosids I | Moraceae | *Ficus copiosa* | GALL028 | Diptera | Agromyzidae |  | OTHE | OTHER | NOT | MID | F |
|  |  |  |  |  | GALL030 | Insecta |  |  | UNKN | OTHER | NOT | MID | F |
| 18 | WAS | Rosids I | Moraceae | *Ficus wassa* | GALL028 | Diptera | Agromyzidae |  | OTHE | OTHER | NOT | MID | F |
|  |  |  |  |  | GALL145 | Insecta |  |  | UNKN | OTHER | NOT | MID | F |
|  |  |  |  |  | GALL147 | Insecta |  |  | UNKN | LEAF | NOT | MID | F |
|  |  |  |  |  | GALL148 | Insecta |  |  | UNKN | LEAF | NOT | LO | F |
| 19 | CEL | Rosids I | Cannabaceae | *Celtis philippensis* | GALL015 | Hemiptera | Triozidae | *Trioza incrustata* Percy**^2^** | HEMI | LEAF | NOT | MID | T |
|  |  |  |  |  | GALL016 | Hymenoptera | Eurytomidae | *Tenuipetiolus* n. sp. 5 (Gates) | OTHE | OTHER | NOT | MID | F |
|  |  |  |  |  | GALL017 | Hemiptera |  |  | HEMI | LEAF | NOT | LO | T |
|  |  |  |  |  | GALL018 | Hemiptera |  |  | HEMI | LEAF | NOT | LO | T |
| 20 | CLA | Rosids I | Cannabaceae | *Celtis latifolia* | GALL020 | Diptera | Agromyzidae |  | OTHE | LEAF | NOT | LO | F |
|  |  |  |  |  | GALL021 | Diptera | Cecidomyiidae |  | CECI | LEAF | NOT | LO | F |
|  |  |  |  |  | GALL024 | Insecta |  |  | UNKN | OTHER | NOT | MID | F |
|  |  |  |  |  | GALL025 | Insecta |  |  | UNKN | LEAF | NOT | LO | T |
|  |  |  |  |  | GALL163 | Insecta |  |  | UNKN | OTHER | NOT | MID | F |
|  |  |  |  |  | GALL164 | Diptera | Agromyzidae |  | OTHE | OTHER | NOT | MID | F |
| 21 | SRS | Rosids II | Myrtaceae | *Syzygium malaccense* | GALL116 | Hymenoptera | Eulophidae | *Euceratoneura* sp. 1 (Hansson) | OTHE | OTHER | NOT | MID | F |
|  |  |  |  |  | GALL118 | Hemiptera |  |  | HEMI | LEAF | NOT | LO | T |
|  |  |  |  |  | GALL120 | Insecta |  |  | UNKN | LEAF | NOT | LO | F |
| 22 | SRB | Rosids II | Myrtaceae | *Syzygium sp.* | GALL110 | Hymenoptera | Eurytomidae | *Tenuipetiolus* n. sp. 8 (Gates) | OTHE | OTHER | NOT | HI | F |
|  |  |  |  |  | GALL111 | Diptera | Cecidomyiidae |  | CECI | LEAF | NOT | LO | F |
|  |  |  |  |  | GALL112 | Hemiptera | Aleyrodidae |  | HEMI | LEAF | NOT | LO | T |
|  |  |  |  |  | GALL115 | Insecta |  |  | UNKN | LEAF | NOT | MID | F |
| 23 | POM | Rosids II | Sapindaceae | *Pometia pinnata* | GALL085 | Thysanoptera |  |  | OTHE | LEAF | NOT | HI | T |
|  |  |  |  |  | GALL086 | Lepidoptera |  |  | OTHE | COMP | HAIRY | MID | F |
|  |  |  |  |  | GALL087 | Hemiptera | Phacopter-onidae | *Cornegenapsylla pometiae* Malenovský and Percy**^2^** | HEMI | LEAF | NOT | MID | T |
| 24 | KLE | Rosids II | Malvaceae | *Kleinhovia hospita* |  |  |  |  |  |  |  |  |  |
| 25 | TRI | Rosids II | Malvaceae | *Trichospermum pleiostigma* |  |  |  |  |  |  |  |  |  |
| 26 | PSL | Asterids | Rubiaceae | *Psychotria ramuensis* | GALL089 | Diptera | Cecidomyiidae | *Rhopalomyia psychotriae* Kolesik**^1^** | CECI | COMP | NOT | MID | F |
|  |  |  |  |  | GALL090 | Diptera | Cecidomyiidae |  | CECI | LEAF | NOT | LO | F |
|  |  |  |  |  | GALL091 | Diptera | Cecidomyiidae |  | CECI | LEAF | NOT | MID | F |
|  |  |  |  |  | GALL096 | Diptera | Cecidomyiidae |  | CECI | LEAF | NOT | MID | F |
|  |  |  |  |  | GALL184 | Lepidoptera |  |  | OTHE | OTHER | NOT | MID | F |
| 27 | PSM | Asterids | Rubiaceae | *Psychotria micralabastra* | GALL101 | Hemiptera | Aleyrodidae |  | HEMI | LEAF | NOT | LO | T |
|  |  |  |  |  | GALL102 | Diptera | Cecidomyiidae |  | CECI | LEAF | NOT | MID | F |
| 28 | PSS | Asterids | Rubiaceae | *Psychotria micrococca* | GALL106 | Diptera | Cecidomyiidae |  | CECI | LEAF | NOT | MID | F |
|  |  |  |  |  | GALL109 | Hymenoptera | Eulophidae | *Ophelimus* sp. (La Salle) | OTHE | OTHER | NOT | MID | F |
|  |  |  |  |  | GALL194 | Diptera | Cecidomyiidae |  | CECI | LEAF | NOT | HI | F |
|  |  |  |  |  | GALL195 | Diptera | Cecidomyiidae |  | CECI | LEAF | NOT | LO | F |
|  |  |  |  |  | GALL214 | Insecta |  |  | UNKN | LEAF | NOT | LO | F |
| 29 | PAV | Asterids | Rubiaceae | *Pavetta platyclada* | GALL082 | Insecta |  |  | UNKN | LEAF | HAIRY | LO | T |
|  |  |  |  |  | GALL211 | Hemiptera | Aleyrodidae |  | HEMI | LEAF | NOT | LO | T |
| 30 | TAR | Asterids | Rubiaceae | *Tarenna buruensis* | GALL134 | Thysanoptera |  |  | OTHE | LEAF | NOT | MID | T |
|  |  |  |  |  | GALL135 | Insecta |  |  | UNKN | OTHER | NOT | MID | F |
|  |  |  |  |  | GALL137 | Diptera | Cecidomyiidae |  | CECI | LEAF | NOT | MID | F |
| 31 | TAB | Asterids | Apocynaceae | *Tabernaemontana aurantiaca* | GALL129 | Hemiptera |  |  | HEMI | LEAF | NOT | LO | T |
|  |  |  |  |  | GALL130 | Insecta |  |  | UNKN | LEAF | NOT | LO | F |
| 32 | TBM | Asterids | Apocynaceae | *Tabernaemontana panacaqui* |  |  |  |  |  |  |  |  |  |

**^1^** Kolesik, P. & Butterill, P.T. 2015. New gall midges (Diptera: Cecidomyiidae) from Papua New Guinea. *Austral Entomology,* 54, 79-86

**^2^** Percy, Diana M., Butterill, Philip T. & Malenovský, Igor (2016) Three new species of gall-forming psyllids (Hemiptera: Psylloidea) from Papua New Guinea, with new records and notes on related species. *Journal of Natural History*, **50**:17-18, 1073-1101, DOI:10.1080/00222933.2015.1104394

**^3^** CECI=Cecidomyiidae, HEMI=Hemiptera, UNKN=Unknown insect, OTHE=Known taxa other than Cecidomyiidae and Hemiptera

**^4^** LEAF=Leaf gall, COMP=complex of multiple modules, OTHER=Non-leaf galls

**^5^** HAIRY=Hairy, NOT=Not hairy

**^6^** LO=low apparency, MID=Medium apparency, HI=High apparency

**^7^** F=False, T=True

## Table S2.

Parasitoid morphospecies (Hymenoptera) with identifier names.

| **sp_code** | **superfamily** | **family** | **genus** | **species** | **identifier** |
| --- | --- | --- | --- | --- | --- |
| CERP001 | Ceraphronoidea | Ceraphronidae |  | sp. 1 | Butterill, P.T. |
| CERP003 | Ceraphronoidea | Ceraphronidae |  | sp. 2 | Gibson, G. |
| MEGA001 | Ceraphronoidea | Megaspilidae |  | sp. 1 | Butterill, P.T. |
| APHE001 | Chalcidoidea | Aphelinidae |  | sp. 1 | Butterill, P.T. |
| APHE002 | Chalcidoidea | Aphelinidae |  | sp. 2 | Butterill, P.T. |
| ENCY001 | Chalcidoidea | Encyrtidae |  | sp. 1 | Butterill, P.T. |
| EULO003 | Chalcidoidea | Eulophidae |  | sp. 1 | Butterill, P.T. |
| EULO005 | Chalcidoidea | Eulophidae |  | sp. 2 | Butterill, P.T. |
| EULO036 | Chalcidoidea | Eulophidae |  | sp. 3 | Butterill, P.T. |
| EULO058 | Chalcidoidea | Eulophidae |  | sp. 5 | Gibson, G. |
| EULO059 | Chalcidoidea | Eulophidae |  | sp. 6 | Gibson, G. |
| EULO047 | Chalcidoidea | Eulophidae | Aprostocetus | s10 | Hansson, C. |
| EULO002 | Chalcidoidea | Eulophidae | Aprostocetus | sp1 | Hansson, C. |
| EULO010 | Chalcidoidea | Eulophidae | Aprostocetus | sp11 | Hansson, C. |
| EULO055 | Chalcidoidea | Eulophidae | Aprostocetus | sp12 | Hansson, C. |
| EULO016 | Chalcidoidea | Eulophidae | Aprostocetus | sp13 | Hansson, C. |
| EULO015 | Chalcidoidea | Eulophidae | Aprostocetus | sp14 | Hansson, C. |
| EULO025 | Chalcidoidea | Eulophidae | Aprostocetus | sp15 | Hansson, C. |
| EULO009 | Chalcidoidea | Eulophidae | Aprostocetus | sp16 | Hansson, C. |
| EULO037 | Chalcidoidea | Eulophidae | Aprostocetus | sp17 | Hansson, C. |
| EULO045 | Chalcidoidea | Eulophidae | Aprostocetus | sp2 | Hansson, C. |
| EULO018 | Chalcidoidea | Eulophidae | Aprostocetus | sp3 | Hansson, C. |
| EULO020 | Chalcidoidea | Eulophidae | Aprostocetus | sp4 | Hansson, C. |
| EULO019 | Chalcidoidea | Eulophidae | Aprostocetus | sp5 | Hansson, C. |
| EULO032 | Chalcidoidea | Eulophidae | Aprostocetus | sp6 | Hansson, C. |
| EULO024 | Chalcidoidea | Eulophidae | Aprostocetus | sp7 | Hansson, C. |
| EULO056 | Chalcidoidea | Eulophidae | Aprostocetus | sp8 | Hansson, C. |
| EULO057 | Chalcidoidea | Eulophidae | Aprostocetus | sp9 | Hansson, C. |
| EULO035 | Chalcidoidea | Eulophidae | Asecodes | sp1 | Hansson, C. |
| EULO031 | Chalcidoidea | Eulophidae | Asecodes | sp2 | Hansson, C. |
| EULO051 | Chalcidoidea | Eulophidae | Baryscapus | sp1 | Hansson, C. |
| EULO044 | Chalcidoidea | Eulophidae | Chrysonotomyia? | sp12 | Hansson, C. |
| EULO001 | Chalcidoidea | Eulophidae | Chrysonotomyia | sp1 | Hansson, C. |
| EULO053 | Chalcidoidea | Eulophidae | Chrysonotomyia | sp10 | Hansson, C. |
| EULO054 | Chalcidoidea | Eulophidae | Chrysonotomyia | sp11 | Hansson, C. |
| EULO006 | Chalcidoidea | Eulophidae | Chrysonotomyia | sp2 | Hansson, C. |
| EULO034 | Chalcidoidea | Eulophidae | Chrysonotomyia | sp3 | Hansson, C. |
| EULO013 | Chalcidoidea | Eulophidae | Chrysonotomyia | sp4 | Hansson, C. |
| EULO008 | Chalcidoidea | Eulophidae | Chrysonotomyia | sp5 | Hansson, C. |
| EULO049 | Chalcidoidea | Eulophidae | Chrysonotomyia | sp6 | Hansson, C. |
| EULO033 | Chalcidoidea | Eulophidae | Chrysonotomyia | sp7 | Hansson, C. |
| EULO048 | Chalcidoidea | Eulophidae | Chrysonotomyia | sp8 | Hansson, C. |
| EULO029 | Chalcidoidea | Eulophidae | Chrysonotomyia | sp9 | Hansson, C. |
| EULO046 | Chalcidoidea | Eulophidae | Closterocerus | sp1 | Hansson, C. |
| EULO041 | Chalcidoidea | Eulophidae | Closterocerus | sp2 | Hansson, C. |
| EULO026 | Chalcidoidea | Eulophidae | Closterocerus | sp3 | Hansson, C. |
| ELAS001 | Chalcidoidea | Eulophidae | Elasmus | sp1 | Hansson, C. |
| EULO042 | Chalcidoidea | Eulophidae | Omphalentedon | sp1 | Hansson, C. |
| EULO023 | Chalcidoidea | Eulophidae | Pediobius | sp1 | Hansson, C. |
| EULO028 | Chalcidoidea | Eulophidae | Piekna | nitens (Boucek, 1988) | Hansson, C. |
| EULO043 | Chalcidoidea | Eulophidae | Zaommomyiella | sp1 | Hansson, C. |
| EULO017 | Chalcidoidea | Eulophidae | Zaommomyiella | sp2 | Hansson, C. |
| EUPE005 | Chalcidoidea | Eupelmidae | Eupelmus | sp1 | Gibson, G. |
| EUPE001 | Chalcidoidea | Eupelmidae | Eupelmus | sp1 | Gibson, G. |
| EUPE004 | Chalcidoidea | Eupelmidae | Eupelmus | sp2 | Gibson, G. |
| EUPE010 | Chalcidoidea | Eupelmidae | Neanastatus | sp2 | Gibson, G. |
| EUPE002 | Chalcidoidea | Eupelmidae | Neanastatus | sp3 | Gibson, G. |
| EUPE006 | Chalcidoidea | Eupelmidae | Reikosiella | n.sp1 | Gibson, G. |
| EUPE008 | Chalcidoidea | Eupelmidae | Reikosiella | n.sp2 | Gibson, G. |
| EUPE007 | Chalcidoidea | Eupelmidae | Reikosiella | n.sp3 | Gibson, G. |
| EUPE003 | Chalcidoidea | Eupelmidae | Reikosiella | n.sp4 | Gibson, G. |
| EURY013 | Chalcidoidea | Eurytomidae |  | sp. 1 | Butterill, P.T. |
| EURY005 | Chalcidoidea | Eurytomidae | Mangoma | n. sp. 1 | Gates, M. |
| EURY014 | Chalcidoidea | Eurytomidae | Phylloxeroxenus | n. sp. 1 | Gates, M. |
| EURY015 | Chalcidoidea | Eurytomidae | Phylloxeroxenus | n. sp. 2 | Gates, M. |
| EURY007 | Chalcidoidea | Eurytomidae | Phylloxeroxenus | n. sp. 3 | Gates, M. |
| EURY021 | Chalcidoidea | Eurytomidae | Phylloxeroxenus | n. sp. 4 | Gates, M. |
| EURY011 | Chalcidoidea | Eurytomidae | Phylloxeroxenus | n. sp. 4 | Gates, M. |
| EURY001 | Chalcidoidea | Eurytomidae | Tenuipetiolus | n. sp. 1 | Gates, M. |
| EURY009 | Chalcidoidea | Eurytomidae | Tenuipetiolus | n. sp. 3 | Gates, M. |
| EURY020 | Chalcidoidea | Eurytomidae | Tenuipetiolus | n. sp. 4 | Gates, M. |
| EURY019 | Chalcidoidea | Eurytomidae | Tenuipetiolus | n. sp. 7 | Gates, M. |
| TORY001 | Chalcidoidea | Megastigmidae | n. gen. 1 nr. Paramegastigmus | n. sp. 1 | Jansta, P. |
| TORY002 | Chalcidoidea | Megastigmidae | n. gen. 1 nr. Paramegastigmus | n. sp. 2 | Jansta, P. |
| MYMA001 | Chalcidoidea | Mymaridae |  | sp. 1 | Butterill, P.T. |
| MYMA004 | Chalcidoidea | Mymaridae |  | sp. 3 | Butterill, P.T. |
| MYMA005 | Chalcidoidea | Mymaridae |  | sp. 4 | Butterill, P.T. |
| MYMA003 | Chalcidoidea | Mymaridae |  | sp. 2 | Butterill, P.T. |
| ORMY001 | Chalcidoidea | Ormyridae | Ormyrus | n. sp. 1 | Gates, M. |
| PERI001 | Chalcidoidea | Perilampidae | Perilampus | sp. 1 | Butterill, P.T. |
| PTRM015 | Chalcidoidea | Pteromalidae |  | sp. 1 | Hansson, C. |
| PTRM001 | Chalcidoidea | Pteromalidae |  | sp. 2 | Butterill, P.T. |
| PTRM010 | Chalcidoidea | Pteromalidae | Cecidelllus | n.sp. | Gibson, G. |
| TETR002 | Chalcidoidea | Tetracampidae | Tetracampe | sp1 | Hansson, C. |
| TETR004 | Chalcidoidea | Tetracampidae | Tetracampe | sp2 | Hansson, C. |
| TETR001 | Chalcidoidea | Tetracampidae | Tetracampe | sp3 | Hansson, C. |
| TRIC001 | Chalcidoidea | Trichogrammatidae |  | sp. 1 | Butterill, P.T. |
| TRIC002 | Chalcidoidea | Trichogrammatidae |  | sp. 2 | Butterill, P.T. |
| BRAC009 | Ichneumonoidea | Braconidae | Spathius (Doryctinae) | dq.2 | Quicke, D. |
| BRAC002 | Ichneumonoidea | Braconidae |  | sp. 4 | Butterill, P.T. |
| BRAC007 | Ichneumonoidea | Braconidae | Spathius (Doryctinae) | dq.1 | Quicke, D. |
| BRAC008 | Ichneumonoidea | Braconidae | Bracon (Braconinae) | dq.6 | Quicke, D. |
| PLAT004 | Platygastroidea | Platygastridae | Inostemma | butterilli (Buhl, 2013) | Buhl, P.N. |
| PLAT006 | Platygastroidea | Platygastridae | Inostemma | convexifrons (Buhl, 2013) | Buhl, P.N. |
| PLAT003 | Platygastroidea | Platygastridae | Inostemma | macarangae (Buhl, 2013) | Buhl, P.N. |
| PLAT005 | Platygastroidea | Platygastridae | Inostemma | novoguineense (Buhl, 2013) | Buhl, P.N. |
| PLAT009 | Platygastroidea | Platygastridae | Inostemma | paramyristicae (Buhl, 2013) | Buhl, P.N. |
| PLAT008 | Platygastroidea | Platygastridae | Platygaster | psychotriae (Buhl, 2013) | Buhl, P.N. |
| PLAT010 | Platygastroidea | Platygastridae | Synopeas | butterilli (Buhl, 2013) | Buhl, P.N. |
| PLAT002 | Platygastroidea | Platygastridae | Synopeas | n. sp. 1 | Buhl, P.N. |
| PLAT007 | Platygastroidea | Platygastridae | Synopeas | psychotriae (Buhl, 2013) | Buhl, P.N. |
| PLAT001 | Platygastroidea | Platygastridae | Synopeas | pterocarpi (Buhl, 2013) | Buhl, P.N. |

**Table S3. Coefficient estimates for focal negative binomial GLMs of parasitoid species richness. The AIC / explained-deviance comparison of candidate models is now Table 2 in the main text. Ordered factors use polynomial contrasts (.L = linear, .Q = quadratic).**

| Model | Term | Estimate | SE | z | P | Notes |
| --- | --- | --- | --- | --- | --- | --- |
| Final: apparency + taxon + log(host gall abundance) | (Intercept) | -1.298 | 0.553 | -2.35 | 0.019 | AIC 246.83; e.d. 50.2% |
| Final: apparency + taxon + log(host gall abundance) | apparency.L | 0.805 | 0.260 | 3.09 | 0.002 |  |
| Final: apparency + taxon + log(host gall abundance) | apparency.Q | -0.072 | 0.206 | -0.35 | 0.726 |  |
| Final: apparency + taxon + log(host gall abundance) | former2HEMI | -0.870 | 0.390 | -2.23 | 0.026 | vs CECI |
| Final: apparency + taxon + log(host gall abundance) | former2OTHE | -0.701 | 0.343 | -2.04 | 0.041 | vs CECI |
| Final: apparency + taxon + log(host gall abundance) | former2UNKN | -1.032 | 0.444 | -2.33 | 0.020 | vs CECI |
| Final: apparency + taxon + log(host gall abundance) | log(host gall abundance) | 0.502 | 0.111 | 4.53 | <0.001 | LRT apparency P=0.008671; taxon P=0.013304 |
| Size sensitivity: size + taxon + log(host gall abundance) | (Intercept) | -1.358 | 0.569 | -2.39 | 0.017 | AIC 248.46; e.d. 48.8% |
| Size sensitivity: size + taxon + log(host gall abundance) | size.L | 0.671 | 0.321 | 2.09 | 0.037 |  |
| Size sensitivity: size + taxon + log(host gall abundance) | size.Q | -0.382 | 0.215 | -1.78 | 0.076 |  |
| Size sensitivity: size + taxon + log(host gall abundance) | former2HEMI | -0.804 | 0.419 | -1.92 | 0.055 |  |
| Size sensitivity: size + taxon + log(host gall abundance) | former2OTHE | -0.935 | 0.388 | -2.41 | 0.016 |  |
| Size sensitivity: size + taxon + log(host gall abundance) | former2UNKN | -1.373 | 0.450 | -3.05 | 0.002 |  |
| Size sensitivity: size + taxon + log(host gall abundance) | log(host gall abundance) | 0.504 | 0.114 | 4.43 | <0.001 | Weaker than apparency final model |

**Table S4. Cecidomyiidae-only negative binomial GLMs for parasitoid species richness (n = 32). Plant module is retained with apparency after simplification, whereas it was not retained in the global model (main-text Table 2). Formerly presented as Table S5 in the submitted supplement.**

| Model / parameters | d.f. | Deviance | AIC | e.d. (%) | P / notes |  |
| --- | --- | --- | --- | --- | --- | --- |
| Full: apparency + open + plant module + surface + log(abundance) | — | — | 142.5 | — | Starting model |  |
| Final: apparency + plant module | 27 | 38.8 | 138.24 | 47.4 | apparency P=2.5e-05; plant module P=0.0009481 |  |
| Coefficient: (Intercept) | — | — | 2.184 | — | SE 0.385; P<0.001 |  |
| Coefficient: apparency.L | — | — | 1.232 | — | SE 0.295; P<0.001 |  |
| Coefficient: apparency.Q | — | — | 0.078 | — | SE 0.255; P=0.759 |  |
| Coefficient: plant_moduleLEAF | — | — | -1.440 | — | SE 0.408; P<0.001 (vs COMP) |  |
| Coefficient: plant_moduleOTHER | — | — | -0.761 | — | SE 0.488; P=0.119 (vs COMP) |  |

**Figure S1.** Boxplots of parasitoid species diversity for various categorical gall traits: a) apparency (see also Table 3), b) openness, c) galled module (COMP = complex: multiple module types galled, LEAF = leaves; OTHER = non-leaf modules), d) hairiness, and e) gall-forming taxon (CECI = Cecidomyiidae, HEMI = Hemiptera, OTHE = Others, UNKN = Unknown). *P-*values are the results of Wilcoxon rank-sum tests (for 2-level traits: b, d) or Kruskal-Wallis tests (for 3- & 4-level traits: a, c, e). Significant results are shown in bold.

**Figure S2.** Tripartite diagram showing discrete compartments in different colours. Coloured compartments convey encapsulate all three levels (plants, galls, & parasitoids). Grey nodes reflect multiple compartments comprising nodes from one or two levels (no parasitoids)
