# Supplementary material for: Gall Apparency Drives Parasitoid Richness in a Highly Specialised Gall‐Parasitoid Food Web From a Tropical Rainforest
Source: Ecol Evol. 2026 Oct 6;16(10):e74375. doi: 10.1002/ece3.74375 (PMC13640053; doi:10.1002/ece3.74375)
Supplement: Supplementary file 1 [file ECE3-16-e74375-s001.zip › manuscriptTransformation.html]

xml version="1.0" encoding="UTF-8" standalone="no"?

01\_MS\_revised\_CLEAN


# **Gall apparency drives parasitoid richness in a highly specialised gall-parasitoid food web from a tropical rainforest**

Philip T. Butterill1, Sam Finnie1,2,3\*, Katerina Sam1,2, Tom M. Fayle1,4, Inga Freiberga1, & Vojtech Novotny1,2,5

1 Biology Centre of the Czech Academy of Sciences, Institute of Entomology, Branisovska 31, 370 05 Ceske Budejovice, Czech Republic

2 University of South Bohemia, Faculty of Science, Branisovska 1760, 370 05 Ceske Budejovice, Czech Republic

3 Xishuangbanna Tropical Botanical Garden, Chinese Academy of Sciences, Menglun, Mengla County, Yunnan 666303, China

4 School of Biological and Behavioural Sciences, Queen Mary University of London, London, UK

5 The New Guinea Binatang Research Centre, PO Box 604, Madang, Papua New Guinea

\*Correspondence: Xishuangbanna Tropical Botanical Garden, Chinese Academy of Sciences, Menglun, Mengla County, Yunnan 666303, China.

E-mail: sam.c.finnie@gmail.com

# **Abstract**

Understanding the complexity of interactions in quantitative terrestrial food webs is a key challenge in ecology, particularly in tropical habitats where reports are scarce. Documenting tri‑trophic interactions in these ecosystems is essential to advance understanding of how community dynamics and specialisation shape network structure. Here we present the largest and most comprehensive plant–gall–parasitoid network to date.

We sampled galls and reared parasitoids from three sites in lowland rainforest near Madang, Papua New Guinea, and combined them into a single quantitative tri‑trophic network. Network statistics were calculated, and gall traits (taxon, apparency, openness, plant module, and pubescence) were tested as predictors of parasitoid richness. We also analysed the potential for apparent competition (PAC) among galls.

The network comprised 32 host plant species, 78 galling insect species, and 102 parasitoid species represented by 820 individuals. Monophagous species dominated both gallers and parasitoids, producing high specialisation (H2’ = 0.99 for plant–gall and 0.90 for gall–parasitoid subwebs). Gall apparency and gall‑forming taxon significantly predicted parasitoid assemblage size: conspicuous galls formed by Cecidomyiidae hosted more parasitoid species. One third of gall species shared no parasitoids, and the remainder showed very low PAC, suggesting limited interspecific enemy‑mediated interactions but potential for intraspecific density dependence.

The existence of highly specialised tri trophic food webs in tropical rainforests has been questioned, given the challenges posed by extreme specificity. Such networks require parasitoids specialised to herbivores that are themselves restricted to single plant species, raising doubts about their feasibility in diverse ecosystems. Our study demonstrates that these networks do occur, with gall forming insects and their parasitoids providing clear evidence of highly specialised tri trophic interactions in tropical forests. We also discuss the potential existence of a latitudinal gradient in gall‑parasitoid specialisation.

# **Introduction**

Studies of food webs have proliferated in recent years (Ings et al., 2009), culminating in a wealth of empirical networks (de Araújo & Maia, 2021; Hrcek et al., 2013; Kaartinen & Roslin, 2011), meta-analyses (Morris et al., 2014; Poisot et al., 2011) and metrics that measure various characteristics of web structure (Blüthgen et al., 2006; Dormann et al., 2009) Food webs provide the framework on which to reconcile the structure of biodiversity with ecosystem function. In this context, tri-trophic networks, comprised of interactions on three trophic levels, are particularly valuable. They provide a comprehensive view of ecosystem dynamics by incorporating the effects of indirect interactions and both bottom-up and top-down control mechanisms. Understanding these interactions can provide valuable insights into the mechanisms driving biodiversity, thereby enhancing our ability to predict and manage changes in ecosystem dynamics (Thompson et al., 2012).

Specialisation is a crucial parameter in network ecology, as it determines the distribution of food resources among species and shapes the potential for indirect interactions and apparent competition within ecological networks. The degree of specialisation in species is influenced by various ecological and evolutionary factors, including evolutionary history, ecological niches, and interspecific interactions. Temporal stability, particularly in tropical regions, has been hypothesised to promote specialisation through stable population dynamics and extended periods of co-evolution (Schleuning et al., 2012).

Insects provide a striking example of the diversity in dietary specialisation, shaped by a variety of ecological and evolutionary pressures. Evolutionary adaptations allow insects to exploit specific resources, leading to the development of specialised feeding modes and behaviours (Hardy et al., 2020). Herbivorous insects, for instance, evolve mechanisms to counteract plant defences, resulting in co-evolutionary dynamics (Loxdale et al., 2011). Competition and natural enemy pressures further drive niche differentiation, promoting specialisation and enabling species to exploit distinct resources or habitats (Britton & Andreou, 2016). The feasibility of highly specialised tri-trophic networks in tropical systems remains uncertain. High species diversity and the complexity of interactions in such ecosystems may constrain the maintenance of extreme specialisation, as these conditions pose significant challenges to the functionality of these networks (Pardikes et al., 2018). Understanding the factors that drive specialisation in insects is therefore key to elucidating the structure and function of ecological networks.

Gallers represent one of the most specialised insect herbivore guilds (Price et al., 1987). They have the unique ability to manipulate plant tissues and induce gall formation (Miller III & Raman, 2019). The close association between galling insects and their host plants leads to highly specialised interactions between these organisms (Araújo et al., 2019). This relationship extends to their interactions with parasitoids. The plant tissues manipulated to form galls provide a distinct ecological niche for parasitoids. However, despite the ubiquity of gall-forming insects, studies focusing on gall-parasitoid networks are still relatively rare, particularly in tropical regions (Cagnolo, 2018).

The degree of specialisation among parasitoids on their galling hosts varies considerably. In temperate regions, parasitoids typically exploit two or more host species rather than being strictly monophagous. This pattern is particularly evident in studies where sampling was confined to a single genus of host plants (Chust et al., 2007; Maldonado-Lopez et al., 2023) or to a limited taxonomic range of gall hosts (Hawkins & Goeden, 1984; Nyman et al., 2007; Rott & Godfray, 2000; Schönrogge et al., 2000). However, when a greater diversity of host plants is sampled, monophagous parasitoids appear to be more prominent (Dawah et al., 1995; Memmott et al., 1994; Tscharntke et al., 2001), especially in the tropics (de Araújo & Maia, 2021; López-Núñez et al., 2019; Luz et al., 2021; Paniagua et al., 2009). Although latitudinal trends in the host specificity of gall parasitoids have not been explicitly studied, a previous study examining multiple host guilds, including gallers, found no consistent trends in parasitoid specificity across latitudes (Morris et al., 2014). Consequently, further research on host–parasitoid interactions in tropical systems are required to resolve how ecological and geographic context shape parasitoid host specificity

Another factor that may alter the parasitoid assemblages of gall forming insects is the specific part of the plant, such as a leaf or stem, where the gall occurs (the “galled plant module”). Gallers often specialise on particular plant modules of the host species. Similar preferences are also exhibited by parasitoids, with galls on certain modules attracting more parasitoid species than others (Hawkins & Gagne, 1989). Another important factor is gall apparency, where a combination of size, structure and colouration of a gall can determine its visual conspicuousness to parasitoids (Joseph et al., 2011). Many galler species produce highly characteristic galls so that it is possible to identify the gall species from the combination of host plant species and gall morphology (Redfern et al., 2002; Russo, 2006). Gall morphology, such as larger, harder, or more elaborate forms, is thought to evolve under top-down parasitoid pressure to prohibit or reduce overall parasitism, as proposed by the Enemy Hypothesis (Price et al., 1987). However, such adaptations do not guarantee enemy-free space (Stone & Schönrogge, 2003). On the contrary, more conspicuous galls have been shown to experience higher parasitism rates and support a greater diversity of parasitoid species (Clouse, 1995; Hawkins & Gagne, 1989), suggesting that visual cues may play a pivotal role for gall parasitoids when locating their hosts (Graziosi, 2015). These findings demonstrate the significance of gall characteristics in shaping parasitoid communities.

The specialisation of higher trophic levels also determines the potential for indirect interactions among lower-level species, such as the potential for apparent competition (PAC). In the context of gall-parasitoid food webs, PAC increases with the proportion of generalists (parasitoids with more than one host species), whereas specialists (parasitoids with a single host species) may impose top-down density-dependent regulation on their hosts. In both cases, the likelihood of an effect depends on the intensity of parasitoid pressure (Frank Van Veen et al., 2006; Morris et al., 2004). While specialist parasitoids reduce the potential for interspecific competition by limiting host overlap, their reliance on a single host species may intensify intraspecific competition, particularly when host populations are constrained. Despite its significance, the degree to which PAC shapes food webs and influences herbivore populations and community structure has seldom been addressed in previous studies.

In this study, we analyse a tri-trophic network from a large plant-gall-parasitoid food web from a tropical Papua New Guinean rainforest. We address the following questions and hypotheses:

1. 1. How specialised are the parasitoids in a community of gall-forming insects in a tropical rainforest system and how does this affect the potential for apparent competition (PAC) between gallers? [H1a] We hypothesise that the majority of parasitoids will be monophagous specialists. Furthermore, we hypothesise that the network will be dominated by specialised parasitoids, driven by the ecological stability of the equatorial location and the high degree of host specialisation among gall-forming insects. However, the extent of this specialisation may be limited by the inherent challenges of maintaining highly specialised networks in a diverse ecosystem. [H1b] The preponderance of specialists within our system will minimise the potential for apparent competition.
2. 2. Do gall characteristics drive parasitoid richness in our system? [H2] We hypothesise that the number of parasitoid species will be significantly influenced by factors such as gall-forming taxa, and galled plant module (the part of plant - leaf, stem, etc. - where the gall occurs). Specifically, we predict that more apparent galls will host more parasitoid species than inconspicuous galls.

# **Methods**

## ***Study sites***

The study was carried out in the vicinity of Madang town in Madang Province, Papua New Guinea (PNG). Sampling took place in the villages of Baitabag, Mis, and Ohu (145°41–7 E, 5°08 –14 S, 0–200 m asl), each of which are surrounded by approximately 200 ha of mixed secondary and primary rainforest. The mean annual rainfall in the Madang area is 3,558 mm with minor seasonal variation and the mean air temperature is 26.5°C, varying little throughout the year (McAlpine, 1983).

## ***Target host plants***

Thirty-two species of locally common woody angiosperms were chosen such that all the major angiosperm clades were represented: basal Eudicots, Rosids I and II, Asterids and a single gymnosperm. The 32 species included 4 species of Ficus and 2 species representing 2 other genera of Moraceae, 3 species of Psychotria and 1 species of Tarenna (Rubiaceae), 3 species of Macaranga and 2 species representing 2 other genera of Euphorbiaceae, 2 species of Tabernaemontana (Apocynaceae), 2 species of Syzygium (Myrtaceae), 2 species of Celtis (Cannabaceae), 2 species representing 2 genera of both Malvaceae and Asparagaceae, 6 species from additional angiosperm families, and a species of Gnetum (Gnetaceae; Gymnosperm) (Table S1). Four plant species did not host any galls and were therefore removed from network analyses in this study. Host plants were selected as locally common woody species and each received equal sampling effort (see Insect sampling); we did not quantify independent spatial availability or community-wide rarity of hosts beyond this equal-effort design.

## ***Insect sampling***

Sampling took place over an eight-month period from August 2010 to March 2011. Sampling effort was recorded as the time spent searching the foliage of each plant species for galls, which was approximately proportional to the leaf area examined. Each plant species was sampled with uniform effort, amounting to 13 sampling events (5 in Mis, and 4 each in Baitabag and Ohu) per species. A single event comprised approximately six hours of foliage searching (3 h each by two collectors) on individuals of the target species. Therefore, total sampling effort per host species was approximately 78 hours (13 events x 6 hours per event). The sample dates of each host species were spread evenly across the sampling period and among sites to mitigate seasonal and site effects experimentally.

Due to the impracticality of counting individual galls, we quantified host gall abundance as the number of galled plant modules. We use "plant module" for the architectural organ on which galls occur, scored in three categories: (i) leaf modules; (ii) non-leaf modules, comprising stems, petioles, flowers, and fruits; and (iii) "complex", when a gall morphospecies occupied more than one organ type. Petioles were classified as non-leaf modules. Galled modules were counted exactly from one to ten and then recorded as 11-100 or >100. This module count is a proxy for gall population size: a single leaf or stem segment may bear multiple galls, so module counts underestimate absolute gall numbers when within-module density is high. We therefore interpret abundance effects conservatively. Only galls formed, or strongly suspected to be formed, by insects were sampled. Fungal, bacterial, and mite galls were ignored.

Galls were morphotyped based on a combination of gall morphology and host plant following (Butterill & Novotny, 2015). We then took photographs and reared the galls in clear plastic “bread” bags for a minimum period of one month. Each rearing bag was filled to no more than one-third capacity with galled plant material to minimise condensation and not impede the observation of emergent insects. We imposed a limit of six rearing bags per morphospecies per sample unit to keep collections to a manageable number, and discarded any excess galled plant material. Rearing bags were checked for emergent insects every 1-2 days.

Parasitoids (Hymenoptera) were sorted to family and then sent to taxonomists for further identification (see Acknowledgements and Table S2 for species lists with identifiers). We did not attempt to distinguish between primary parasitoids and hyperparasitoids in this study and, therefore, our network reflects total parasitism. For brevity, we refer to all morphospecies as “species'' from here on. For the purposes of this study, we categorised all parasitoids that we found to parasitise a single host species as ‘specialists’, and those that we found to parasitise two or more host species as ‘generalists’.

## **Data analyses**

## ***Network statistics***

We measured species level and network level specialisation using the indices d' and H2', respectively, as defined by (Blüthgen et al., 2006). These indices yield values between 0 for extreme generalisation and 1 for extreme specialisation. We used the species-level index, d', to measure the exclusivity or complementarity of interactions. We interpreted this as the “deviation of the actual interaction frequencies from a null model which assumes that all partners are used in proportion to their availability” (Blüthgen et al., 2006). However, this index could potentially underestimate specialisation by treating a rare species (that could be truly specialised) on a commonly visited host as a generalist because it conformed to the null model. We also measured vulnerability, the mean number of parasitoid species per host and number of compartments, the number of subwebs disconnected from the rest of the network (for further details, see (Blüthgen, 2010; Blüthgen et al., 2007; Dormann et al., 2009). Network statistics were calculated in the R package, bipartite (Dormann et al., 2014). A tripartite diagram (Fig. 2) was developed in Python (Python Software Foundation) using the package NetworkX (Hagberg et al., 2008).

## ***Sampling coverage***

Sampling completeness was assessed with species-accumulation and sample-coverage curves. Sampling effort was measured in events (one plant species sampled on one day; 13 events per species). Using incidence-frequency data, we applied the rarefaction and extrapolation framework of Chao et al. (2014), which estimates Hill-number diversity (here species richness, order q = 0) and sample coverage as functions of sampling effort, with bootstrap confidence intervals for interpolated and extrapolated curves. Analyses were implemented in the R package iNEXT (Hsieh et al., 2016) for individual sites and all sites combined.

## ***Indirect interactions***

We calculated the potential for apparent competition using the PAC function of the bipartite package in R (Dormann et al., 2009), following Muller et al. (1999). We created an H x H matrix of standardised values between 0 and 1, dij, where H is the number of host species, and i and j are individual host species. We define dij as the probability that a parasitoid attacking species i developed on species j (Morris et al., 2005). Hence dij indicates the degree to which two host galler species share a parasitoid community, and therefore the degree to which increases in the abundance of one host species have the potential to increase populations of shared parasitoids and decrease the abundance of the second host species. When i and j are the same species (diagonal cells), dij represents apparent intraspecific competition.

## ***Gall traits***

Galls were characterised by appearance, structure, and the taxon of the gall former (Table 1). Size classes were small (<5 mm), mid (5-15 mm), and large (>15 mm). Similarity to the host organ was scored visually as low, mid, or high from photographs and specimens. Apparency (low, mid, or high) was then assigned from the joint size x similarity classification (Fig. 1). Scores reflect human visual assessment under a standardised inspection of vouchers; they do not incorporate ultraviolet reflectance or parasitoid spectral sensitivity. To test whether size alone could replace this composite metric, we fitted parallel models using categorical size (and, separately, ordinal size coded 1-3) in place of apparency (Results; Table 2).

The response variable in all trait models was parasitoid species richness (the number of parasitoid species reared from each gall morphospecies). Host gall abundance (minimum number of galled plant modules) entered only as a covariate, log-transformed to stabilise residuals and reduce leverage of extreme values. We first fitted negative binomial GLMs for each explanatory trait together with the abundance covariate, then a full model containing apparency, gall-former taxon, openness, plant module, surface pubescence, and log(abundance). Backward simplification used AIC and likelihood-ratio tests. Explained deviance was calculated following Zuur et al. (2009). An analogous full model substituting size for apparency provided a sensitivity analysis. We also analysed Cecidomyiidae alone, the dominant gall taxon, to test whether plant-module effects emerge when host lineage is held constant (Table S4). Trait analyses used species-level aggregates because richness and morphological traits are summarised at that level; we therefore used negative binomial GLMs rather than GLMMs with month or site random effects. Model fits were checked with simulated quantile residuals using DHARMa (Hartig, 2024). For the final global model, the size-sensitivity model, and the Cecidomyiidae-only model, tests of residual uniformity, dispersion, and zero inflation were non-significant (all P > 0.10), indicating adequate model fit. Negative binomial GLMs used glm.nb in MASS (Ripley et al., 2013). Model comparison statistics are given in Table 2, with additional coefficient detail in Table S3.

Finally, we produced scatterplots of the number of parasitoid species versus host gall abundance with fitted lines for each trait factor level, using predicted values from the model.

# **Results**

We reared a conservative estimate of 8,150 galled plant modules yielding 820 individual parasitoids of 102 species. Parasitoids were reared from 42 (out of 78) host gall species on 28 (out of 32) host plant species. The parasitoid community was dominated by the superfamily Chalcidoidea (92.4% of individuals and 83.3% of species), followed by Platygastroidea (3.8%, 9.8%), Ichneumonoidea (3.4%, 3.9%), and Ceraphronoidea (0.4%, 2.9%). The family Eulophidae accounted for 46% of all species and 40% of individuals. By traditional definition, 81 of the 102 (79%) parasitoid species in the network were specialists, feeding on only a single host, a figure significantly boosted by the number of rare species observed (37 singletons and 16 doubletons). On removal of singleton parasitoid species, 68% of the remaining species were monophagous (44 of 65), and when doubleton species were also removed, 67% were monophagous (33 of 49). When the complementary specialisation index, d' (Bluthgen et al., 2006), was applied to the parasitoid species in the network, 62 of 102 species (61%) had d' > 0.5. Species-level d' values were significantly greater than the interpretive midpoint of 0.5 (Wilcoxon signed-rank test, V = 3694, P = 0.0004; mean +/- SD = 0.60 +/- 0.26). A binomial test of the proportion exceeding 0.5 was likewise significant (P = 0.037). High specialisation at the species level resulted in a high level of network-wide specialisation (H2' = 0.89).

According to sampling coverage estimates, our parasitoid species represented approximately 95% of the total parasitoid community for the targeted host plants and galls (Fig. 3b). Further sampling to diversity asymptote (Fig. 3a) could potentially increase the parasitoid richness by 10 to 35 species for the 32 plant species sampled, but this would require almost double the sampling effort.

## ***Food web statistics***

In the entire tri-trophic web (Fig. 2), there were six discrete compartments that contained members from each trophic level (Fig. S2). These compartments were highly asymmetric: one large compartment comprised 158 species nodes (~75% of species in the whole network), and the remaining five were small (13, 7, 6, 3 and 3 nodes). In the gall-parasitoid sub-network, there were 17 separate compartments: the largest with 98 species nodes, and sixteen smaller compartments with between 2-6 nodes. Five gall-parasitoid compartments were species pairs with a single exclusive interaction.

## ***Indirect interactions***

The parasitoid overlap graph (Fig. 4) provides a visual representation of the potential for apparent competition (PAC) in the gall-parasitoid food web. Out of a possible 861 heterospecific host gall pairs (42 x 42, minus same-species pairs), only 62 (7%) had non-zero potential (mean PAC of pairs with non-zero values was 0.069 (sd=0.12). Sixteen of the 42 host gall species had zero potential for competition due to not sharing any parasitoids with other species. The mean PAC among same-species pairs (i.e. the diagonal cells of the matrix, representing intraspecific PAC), in contrast, was 0.76 (sd=0.26). In this food web, therefore, there is significantly more potential for intraspecific competition among hosts than for interspecific competition.

## ***Gall traits***

The 78 gall species sampled were formed by Cecidomyiidae (41%), Sternorrhyncha (Hemiptera; 19%), Chalcidoidea (Hymenoptera; 5%), Agromyzidae & Thysanoptera (4% each). Nineteen species (24%) remained unidentified. Only one species was found to gall more than one host plant species, the rest of the species were monophagous (Butterill & Novotny, 2015).

In all GLMs, host gall abundance was the strongest predictor of parasitoid species richness, as expected. Among single-trait models, apparency (explained deviance, e.d. = 42.6%), gall taxon (e.d. = 43.5%), and gall openness (e.d. = 34.6%) improved fit relative to abundance alone (e.d. = 29.2%; Table 2). In the full model with all predictors, only apparency, gall-forming taxon, and host gall abundance were retained after model simplification (Table 2; coefficients in Table S3). The final model explained 50.2% of deviance (AIC = 246.83; likelihood-ratio tests: apparency P = 0.008671, taxon P = 0.013304, abundance P < 0.001). Plant module, surface pubescence, and openness were not retained in the global model. Individual gall traits are visualised in supplementary boxplots (Fig. S1). The result that different gall-forming taxa support parasitoid assemblages of varying richness is driven mainly by the Cecidomyiidae, which hosted more species than the other taxa, although the three agromyzid gallers, grouped with 'others', actually hosted more species than cecidomyiids on average (Fig. 5).

Substituting categorical gall size for apparency produced a weaker final model (AIC = 248.46; e.d. = 48.8%) than the equivalent apparency model (AIC = 246.83; e.d. = 50.2%; Table 2). Ordinal size was likewise inferior to apparency. We therefore retain apparency as the focal morphological predictor while reporting size as a sensitivity analysis.

Restricting the analysis to Cecidomyiidae (n = 32 morphospecies), the dominant gall taxon, changed predictor importance (Table S4). After simplification, apparency and plant module were retained (AIC = 138.24; e.d. = 47.4%), whereas host gall abundance was not. Both apparency (P = 2.5e-05) and plant module (P = 0.0009481) significantly predicted parasitoid richness within Cecidomyiidae. Thus plant module, non-significant in the global model, becomes important once host lineage is held constant, indicating that taxon identity can mask microhabitat-level effects in multi-taxon analyses.

# **Discussion**

## ***Food web structure***

Our study reveals a highly diverse network of plant-gall-parasitoids, marked by significant specialisation. Previous work at these study sites documented a highly specialised system of gall‑forming insects on their host plants (Butterill & Novotny, 2015). Building on this foundation, the present analysis shows that the parasitoids of these gallers also display a reciprocal degree of specialisation. The resulting network is characterised by high compartmentalisation and low connectance, features that substantially limit the potential for indirect interactions. This finding contrasts with the hypothesis that the third trophic level in diverse tropical forests is constrained to be generalist, given the low resource abundance associated with specialist herbivores on rare plant species. For example, Lewis et al. (2002) documented predominantly generalist parasitoid communities in leaf-mining systems in a tropical forest. However, our study demonstrates that highly specialised tri-trophic networks, such as those involving gall-forming insects and their parasitoids, can occur in highly diverse ecosystems.

Highly specialised antagonistic networks are not uncommon in previous studies. However, they are typically characteristic of smaller networks with fewer species or fewer observed interactions. In contrast, our network represents the largest tropical plant-gall-parasitoid network known to date. Morris et al. (2004) demonstrated that host-parasitoid networks had an average specialisation of H2’ = 0.65. This underscores the uniquely high level of specialisation in our network (H2’ = 0.90).

Extremely high levels of specialisation are not ubiquitous in gall-parasitoid networks. For instance, several studies have reported a predominance of generalist parasitoids. Both Chust et al. (2007) in Albera and Maldonado-López et al. (2022) in west-central Mexico found that more than half of the parasitoids in oak forests were generalists. Further, in the Cordoba mountains in Argentina, species richness was higher among specialists (54% of all species) than generalists, but generalists were significantly more abundant, accounting for 83% of parasitoid individuals (Kuzmanich et al., 2023). Along the Portuguese coast, generalists made up 87% of individuals, while 65% of parasitoid species parasitised a single host species (López-Núñez et al., 2019). One clear distinction between these studies and ours is the equatorial location of our site. Studies conducted at high latitudes or altitudes, which have greater seasonal variation, tend to report a higher proportion of generalist parasitoids. In contrast, studies such as ours, conducted in tropical locations closer to the equator, tend to find a higher proportion of specialist parasitoids. For example, (Paniagua et al., 2009) found that 91.2% of parasitoids were specialists in Panama, while (De Araújo & Maia, 2021) and (Luz et al., 2021) reported specialist percentages of 64.3% and 86.7% in southeast and southern Brazil respectively. (Morris et al., 2014) analysed 28 host-parasitoid networks from temperate to tropical locations, encompassing five host guilds (including gallers), and found no evidence that network specialisation increased with decreasing latitude. However, confidence in the lack of a latitudinal trend is hampered by insufficient data, and subsequent studies over the last decade, particularly those focusing on gall-parasitoid networks, are challenging this notion, with our study being no exception.

Consistent with our hypothesis [H1b], interspecific PAC was low, whilst intraspecific PAC was high. Large numbers of specialised parasitoids could be the result of temporal niche availability and host insect voltinism. In gall-parasitoid systems, specialist parasitoids tend to be attackers of early-stage insects, whereas generalists tend to be late-stage (Askew, 1975). Furthermore, there is a trade-off dependent on the chosen strategy, with specialists having high fecundity and low competitive ability ("r" strategists), whereas generalists have low fecundity, but high competitive ability ("K" strategists) allowing them to compete with a wider range of species for limited resources (Askew, 1975; Force, 1974). The large number of specialist parasitoid species in the food web, therefore, may reflect the lack of interspecific competition (Fig. 4) and indicate that resources are plentiful. In temperate regions, most gallers are either uni- or bivoltine, their generations being synchronisd with either or both spring and autumn seasons. Thus, for both specialists and generalists, there are at most two temporal niches per year in which to prosper, leading to high competition for transitory resources. In tropical systems, however, the presence of overlapping generations means that only a small proportion of gallers are at a suitable developmental stage for parasitoid attack at any given time. This dynamic favours specialist parasitoids with life cycles precisely synchronized to their obligate hosts, as opposed to generalists, which typically target the more limited and sporadic late-stage galls.

Another criterion that has not been explicitly tested but appears to influence specialisation in gall-parasitoid networks is gall host taxa. The distribution of galling taxa, particularly the contrast between cynipid and cecidomyiid gallers, likely plays a pivotal role in this dynamic. Cynipids, whose diversity and abundance are highly correlated with the presence of Quercus trees, dominate gall communities in the northern hemisphere, especially at higher latitudes (Maldonado-López et al., 2016). This is due to Quercus trees constituting approximately 87% of cynipid host plants (Abrahamson et al., 2003). However, Quercus is mostly absent from the tropics, resulting in a vastly reduced cynipid fauna (other Fagaceae genera, Lithocarpus and Castanea, can host tropical cynipids; see (Nieves-Aldrey & Butterill, 2014). Additionally, studies have shown that cynipid gallers can be more generalist than cecidomyiids which are almost exclusively host specialist (de Araújo & Maia, 2021). The consequences of such extreme host specialisation for parasitoids have been unclear, but our results show that parasitoid specialisation closely mirrors that of their hosts, indicating strong reciprocal patterns in this system.

Further, evidence for parasitoids of cecidomyiid gallers being highly specialised is supported by the literature. For example, the three quantitative food webs produced by (Paniagua et al., 2009) from a tropical forest in Panama were dominated by monophagous parasitoids of predominantly cecidomyiid hosts. Similarly, studies by (De Araújo & Maia, 2021), and (Luz et al., 2021) observed that the majority of parasitoids were host-specific when sampling exclusively cecidomyiids. This indicates that parasitoids may be more specialised on cecidomyiid hosts, which accounted for approximately 70% of gall taxa in our study.

Due to cecidomyiids dominating tropical galler communities, it remains difficult to disentangle whether parasitoid specialisation reflects latitudinal patterns, taxonomic differences, or other factors. Galling taxa clearly influenced parasitoid richness in our study, but we cannot yet determine how this relates to specialisation. Studies on gall-parasitoid interactions in transitional forests at intermediate northern latitudes, where cynipid and cecidomyiid gallers co‑occur, would provide an ideal setting to test these drivers more directly.

Estimates of parasitoid host specificity are also constrained by sampling bias, particularly in species‑rich tropical systems where many hosts are rare and taxonomic knowledge is limited. Although our sampling focused on the most common tree species, the asymptotic species‑accumulation curves indicate that we captured most gall–parasitoid interactions associated with these hosts (Fig. 3). This represents only part of the local flora, but it offers a robust basis for assessing patterns of host specificity and for guiding broader comparative work. Based on our sampling of 32 plant species, which yielded 102 parasitoid species, we estimate an average of approximately three parasitoid species per plant. If this relationship holds across the more than 500 woody plant species recorded in the nearby Wanang Forest Dynamics Plot (Davies et al., 2021), the total parasitoid diversity in the forest could exceed 1,000 species. Such an estimate points to remarkably high parasitoid diversity, underscoring the complexity and richness of multi-trophic interactions in tropical rainforests. Moreover, the parasitoid–host species ratio (102 parasitoid species across 78 hosts; 1.3 species per host) surpasses reports from other tropical insect guilds. For instance, (Lewis et al., 2002) documented a tri-trophic network centred on leaf miners with 0.9 species per host (84 parasitoid species across 93 hosts), while (Hrcek et al., 2013) reported 0.65 species per host for externally feeding caterpillars (172 parasitoid species across 266 caterpillar hosts) in Papua New Guinea. This may partly reflect the high local densities at which galls are often found, thus providing abundant material for rearing, but also suggests a higher proportion of “dark” diversity among gall formers compared to other groups.

This tri-trophic system is doubly specialised, with both herbivores and their parasitoids often being monophagous. This contrasts with leaf miner and externally feeding caterpillar systems (Hrcek et al., 2013; Lewis et al., 2002), where specialist leaf miners tend to be attacked by generalist parasitoids, and generalist caterpillars by specialist parasitoids. Such extreme specialisation can lead to very low resource densities—for instance, a monophagous galler supporting a monophagous parasitoid.

The high specialisation we observe is unlikely to be a simple geographical coincidence. Several interlocking processes can generate and maintain specialist-dominated gall-parasitoid webs near the equator. First, low climatic seasonality supports overlapping host generations, so that only a fraction of galls are at a suitable developmental stage at any time; this temporal structure favours parasitoids whose life cycles track a single host rather than opportunistic generalists dependent on brief, synchronous resource pulses (Askew, 1975). Second, gallers themselves are overwhelmingly monophagous in this system (Butterill & Novotny, 2015), so reciprocal specialisation at the third trophic level is facilitated by predictable, chemically and morphologically distinct niches. Third, compartmentalisation of the network limits host switching and apparent competition, reinforcing host fidelity once specialised lineages establish. Fourth, taxonomic turnover with latitude, declining cynipid-Quercus systems and increasing cecidomyiid dominance, shifts the galler pool toward hosts whose parasitoids are repeatedly reported as highly host-specific in tropical studies (Paniagua et al., 2009; de Araujo & Maia, 2021; Luz et al., 2021). Together, phenology, reciprocal specialisation, network topology, and galler taxonomic composition provide a mechanistic basis for elevated H2' and specialist frequencies in our equatorial web, beyond a purely latitudinal description.

## ***Gall traits***

Since sampling was standardised across host plants, host gall abundance explained the greatest share of parasitoid species richness in all models. Among morphological predictors, gall apparency and gall-forming taxon were retained globally, whereas openness, plant module, and pubescence were not (Table 2). More apparent galls tended to support more parasitoid species, consistent with H2, but we treat this as a correlative pattern rather than a definitive demonstration that human-perceived apparency causes richer parasitoid assemblages.

Within Cecidomyiidae, plant module predicted parasitoid richness alongside apparency (Table S4; e.d. = 47.4%). Globally, module effects appear masked by differences among galler lineages that occupy different organs; once the analysis is restricted to the dominant lineage, architectural location (leaf versus non-leaf or complex modules) contributes additional signal. Plant module traits may therefore condition parasitoid recruitment primarily within host clades, whereas among-taxon analyses emphasise lineage identity and overall conspicuousness.

In line with our hypothesis [H2], more apparent galls tended to host more parasitoid species. More apparent galls are those that ‘stand out’ from their host plants, usually due to relatively large size, contrasting colours, or both in the most apparent galls. If increased conspicuity of galls leads to increased parasitoid richness, this raises the question of the significance of visual location for parasitoids when finding their host galls. Visual cues have been identified as significant factors in host location in parasitoids in many previous studies. However, these visual cues typically exert a greater influence when combined with chemical cues, one aspect that was beyond the scope of this study. Yet, gall apparency was previously identified as a key factor influencing the size of parasitoid assemblages in cecidomyiids (Hawkins & Gagn�, 1989). Our study supports this finding, with two-thirds of the most apparent galls being formed by cecidomyiids. When we analysed data for cecidomyiids separately, apparency emerged as the most significant predictor of parasitoid richness, surpassing even gall abundance that was dropped from the model (Table S4). This pattern is consistent with a role for visual conspicuousness in shaping parasitoid assemblages of cecidomyiids, while recognising the limitations of human-scored apparency discussed below.

Apparent galls are often those with striking colouration. This distinctive colouration is a result of hijacked plant pigments such as carotenoids, anthocyanins, as well as tannins and other phenolic compounds (Lev-Yadun, 2016). Recently, “the aposematic gall hypothesis” was proposed by (Inbar et al., 2010) suggesting that chemically protected galls, which are also visually conspicuous are aposematic, which could reduce their vulnerability to predators and parasitoids. However, in other host-parasitoid systems, such as caterpillar-parasitoids, it has been shown that aposematism, in caterpillars whilst decreasing predation rates, can actually cause increased parasitism rates and parasitoid richness on aposematic hosts (Finnie et al., 2024). This has been justified with the “safe haven” hypothesis, which postulates that aposematic hosts that are better protected from predators actually provide ideal conditions for parasitoids (Lampert et al., 2010), and it is possible that this theory extends to gall forming hosts. Another possible justification may lie in certain galling species’ having the ability to silence or suppress parasitoid-attracting compounds during gall formation, which could emphasise the importance of using visual cues instead for host location (Tooker et al., 2008). While our study does not explicitly test these theories, it underscores the need for further targeted research to understand the mechanisms driving higher parasitoid richness in more apparent galls. This research should also consider that 'apparency' in this context is based on human perception, which may not fully reflect ecological realities for gall parasitoids. Future studies should focus on the significance and justification of visual cues for gall parasitoids in both tropical and temperate locations. Importantly, our discrete apparency scale was not psychophysically validated against parasitoid vision (including ultraviolet reflectance) or under natural canopy lighting, and observer scores necessarily reflect human perception. Although size alone did not outperform apparency in AIC comparisons (Table 2), we refrain from strong causal claims that apparency per se structures the network. Future studies should quantify size continuously, measure spectral reflectance (including UV), and test host-location behaviour under realistic lighting.

## ***Conclusions***

In this study, we unveil a highly diverse tri-trophic plant-insect food web from a tropical forest, characterised by the unique interaction between highly specialised galling herbivores and their specialised parasitoids. Our findings indicate a marked prevalence of specialist parasitoids in our gall-parasitoid food web. After comparing our findings with existing literature, we tentatively propose the potential existence of a latitudinal gradient in the specialisation of gall-parasitoid food webs, with specialisation intensifying towards the equator. This gradient may be influenced, in part, by a shift in the dominant galling taxa towards the tropics, with cecidomyiid gallers hosting more specialised parasitoid assemblages than cynipids. Additionally, our study reveals that galls with greater visual conspicuousness host a richer diversity of parasitoids. This correlative pattern is consistent with a contribution of visual conspicuousness to parasitoid richness, pending functional validation of apparency cues. Overall, our research documents a highly specialised plant–galler–parasitoid tri‑trophic food web from a tropical rainforest and provides new insights into its structure and diversity.

## ***Acknowledgements***

Acknowledgements: The authors would like to extend huge thanks to the hard-working collectors involved in this project from the local communities: Aisak Kutil, Gunn & Michael (Mis), Nataniel Mataton, Basil & Tara (Baitabag), and Austin & Jafet (Ohu); and all staff at the New Guinea Binatang Research Centre, especially: Martin Keltim, Thomas Pius, Ismael Kiatik, and Brus Isua. We dedicate this work to the memory of Aisak Kutil, who passed away in 2017. For the identification of parasitoid morphospecies, we express our sincere thanks to Christer Hansson (Chalcidoidea: Eulophidae and Tetracampidae), Gary Gibson (Chalcidoidea: Eupelmidae and Pteromalidae), Mike Gates (Chalcidoidea: Eurytomidae and Ormyridae), Petr Jansta (Chalcidoidea: Torymidae), Donald Quicke (Ichneumonoidea: Braconidae), and Peter Buhl (Platygastroidea). We also thank the CAS Praemium Academiae grant to VN, GACR grant 23-07776S (to VN), ERC Starting grant 805189 (to KS), and Czech Science Foundation 22-17593M (to KS)

## ***Conflict of interest***

Conflict of interest: The authors declare no competing interests.

## ***Author contributions***

Author contributions: Philip T. Butterill and Vojtech Novotny conceived the ideas and designed the methodology; Philip T. Butterill, with assistance from Inga Freiberga, collected and reared specimens and assembled the dataset; Philip T. Butterill and Sam Finnie analysed the data; Philip T. Butterill and Sam Finnie led the writing of the manuscript. Tom M. Fayle, Katerina Sam, Inga Freiberga and Vojtech Novotny contributed substantially to the final version of the manuscript. All authors contributed critically to the drafts and gave final approval for publication.

## ***Data availability***

Data availability: All the required data are uploaded as supplementary material.

# **References**

Abrahamson, W. G., Hunter, M. D., Melika, G., & Price, P. W. (2003). Cynipid gall-wasp communities correlate with oak chemistry. Journal of Chemical Ecology, 29(1), 209–223. https://doi.org/10.1023/A:1021993017237

Araújo, W. S. de, Freitas, É. V. D. de, Kollár, J., Pessoa, R. O., Corgosinho, P. H. C., Valério, H. M., Falcão, L. A. D., Fagundes, M., Pimenta, M. A. S., & Faria, M. L. de. (2019). Host specialization in plant-galling interactions: Contrasting mites and insects. Diversity, 11(10), 180.

Askew, R. R. (1975). The Organisation of Chalcid-Dominated Parasitoid Communities Centred upon Endophytic Hosts. In P. W. Price (Ed.), Evolutionary Strategies of Parasitic Insects and Mites (pp. 130–153). Springer US. https://doi.org/10.1007/978-1-4615-8732-3\_7

Blüthgen, N. (2010). Why network analysis is often disconnected from community ecology: A critique and an ecologist’s guide. Basic and Applied Ecology, 11(3), 185–195.

Blüthgen, Nico, Menzel, F., & Blüthgen, Nils. (2006). Measuring specialization in species interaction networks. BMC Ecology, 6(1), 9. https://doi.org/10.1186/1472-6785-6-9

Blüthgen, Nico, Menzel, F., Hovestadt, T., Fiala, B., & Blüthgen, Nils. (2007). Specialization, constraints, and conflicting interests in mutualistic networks. Current Biology, 17(4), 341–346.

Britton, J. R., & Andreou, D. (2016). Parasitism as a driver of trophic niche specialisation. Trends in Parasitology, 32(6), 437–445.

Butterill, P. T., & Novotny, V. (2015). Gall-forming insects in a lowland tropical rainforest: Low species diversity in an extremely specialised guild. Ecological Entomology, 40(4), 409–419. https://doi.org/10.1111/een.12198

Cagnolo, L. (2018). The Future of Ecological Networks in the Tropics. In W. Dáttilo & V. Rico-Gray (Eds.), Ecological Networks in the Tropics: An Integrative Overview of Species Interactions from Some of the Most Species-Rich Habitats on Earth (pp. 171–183). Springer International Publishing. https://doi.org/10.1007/978-3-319-68228-0\_12

Chao, A., Gotelli, N. J., Hsieh, T. C., Sander, E. L., Ma, K. H., Colwell, R. K., & Ellison, A. M. (2014). Rarefaction and extrapolation with Hill numbers: A framework for sampling and estimation in species diversity studies. Ecological Monographs, 84(1), 45–67. https://doi.org/10.1890/13-0133.1

Chust, G., Garbin, L., & Pujade-Villar, J. (2007). Gall wasps and their parasitoids in cork oak fragmented forests. Ecological Entomology, 32(1), 82–91. https://doi.org/10.1111/j.1365-2311.2006.00850.x

Clouse, R. M. (1995). The Importance of Gall Size for Sea Daisy Gall Midge Parasitoids. Journal of the Kansas Entomological Society, 68(2), 184–188.

Davies, S. J., Abiem, I., Salim, K. A., Aguilar, S., Allen, D., Alonso, A., Anderson-Teixeira, K., Andrade, A., Arellano, G., & Ashton, P. S. (2021). ForestGEO: Understanding forest diversity and dynamics through a global observatory network. Biological Conservation, 253, 108907.

Dawah, H. A., Hawkins, B. A., & Claridge, M. F. (1995). Structure of the parasitoid communities of grass-feeding chalcid wasps. Journal of Animal Ecology, 708–720.

de Araújo, W. S., & Maia, V. C. (2021). Topological structure of a tritrophic network composed of host plants, gall-inducing insects and parasitoids in a restinga area in Brazil. Entomological Science, 24(2), 201–216. https://doi.org/10.1111/ens.12468

De Araújo, W. S., & Maia, V. C. (2021). Topological structure of a tritrophic network composed of host plants, gall‐inducing insects and parasitoids in a restinga area in Brazil. Entomological Science, 24(2), 201–216. https://doi.org/10.1111/ens.12468

Dormann, C. F., Fruend, J., Gruber, B., Dormann, M. C. F., LazyData, T., & ByteCompile, T. (2014). Package ‘bipartite.’ Visualizing Bipartite Networks and Calculating Some (Ecological) Indices (Version 2.04).(R Foundation for Statistical Computing.) Available at Https://Cran. r-Project. Org/Web/Packages/Bipartite/Index. Html [Verified 28 July 2015]. http://download.nust.na/pub3/cran/web/packages/bipartite/bipartite.pdf

Dormann, C., Frund, J., Bluthgen, N., & Gruber, B. (2009). Indices, Graphs and Null Models: Analyzing Bipartite Ecological Networks. Open Journal of Ecology, 2, 7–24. https://doi.org/10.2174/1874213000902010007

Finnie, S., Butterill, P., Novotny, V., Redmond, C., Jorge, L. R., Abe, T., Lamarre, G. P. A., Maicher, V., & Sam, K. (2024). Vertical stratification and defensive traits of caterpillars against parasitoids in a lowland tropical forest in Cameroon. Oecologia. https://doi.org/10.1007/s00442-024-05542-x

Force, D. C. (1974). Ecology of Insect Host-Parasitoid Communities: Competition may be the most vital factor in the structure and function of these systems. Science, 184(4137), 624–632. https://doi.org/10.1126/science.184.4137.624

Frank Van Veen, F. J., Morris, R. J., & Godfray, H. C. J. (2006). APPARENT COMPETITION, QUANTITATIVE FOOD WEBS, AND THE STRUCTURE OF PHYTOPHAGOUS INSECT COMMUNITIES. Annual Review of Entomology, 51(1), 187–208. https://doi.org/10.1146/annurev.ento.51.110104.151120

Graziosi, I. (2015). Invasiveness of an exotic gall wasp is influenced by interactions with exotic and endemic organisms. https://uknowledge.uky.edu/entomology\_etds/15/

Hagberg, A., Swart, P., & S Chult, D. (2008). Exploring network structure, dynamics, and function using NetworkX. Los Alamos National Lab.(LANL), Los Alamos, NM (United States). https://www.osti.gov/biblio/960616

Hardy, N. B., Kaczvinsky, C., Bird, G., & Normark, B. B. (2020). What We Don’t Know About Diet-Breadth Evolution in Herbivorous Insects. Annual Review of Ecology, Evolution, and Systematics, 51(1), 103–122. https://doi.org/10.1146/annurev-ecolsys-011720-023322

Hawkins, B. A., & Gagn�, R. J. (1989). Determinants of assemblage size for the parasitoids of Cecidomyiidae (Diptera). Oecologia, 81(1), 75–88. https://doi.org/10.1007/BF00377013

Hawkins, B. A., & Goeden, R. D. (1984). Organization of a parasitoid community associated with a complex of galls on Atriplex spp. In southern California. Ecological Entomology, 9(3), 271–292. https://doi.org/10.1111/j.1365-2311.1984.tb00851.x

Hrcek, J., Miller, S. E., Whitfield, J. B., Shima, H., & Novotny, V. (2013). Parasitism rate, parasitoid community composition and host specificity on exposed and semi-concealed caterpillars from a tropical rainforest. Oecologia, 173, 521–532.

Hsieh, T. C., Ma, K. H., Chao, A., & Hsieh, M. T. (2016). Package ‘iNEXT’. Interpolation and Extrapolation for Species Diversity. URL Http://Chao Stat Nthu Edu Tw/Blog/Software-Download/(Accessed 228 2017). https://mirror.las.iastate.edu/CRAN/web/packages/iNEXT/iNEXT.pdf

Inbar, M., Izhaki, I., Koplovich, A., Lupo, I., Silanikove, N., Glasser, T., Gerchman, Y., Perevolotsky, A., & Lev-Yadun, S. (2010). Why do many galls have conspicuous colors? A new hypothesis. Arthropod-Plant Interactions, 4(1), 1–6. https://doi.org/10.1007/s11829-009-9082-7

Joseph, M. B., Gentles, M., & Pearse, I. S. (2011). The parasitoid community of Andricus quercuscalifornicus and its association with gall size, phenology, and location. Biodiversity and Conservation, 20(1), 203–216. https://doi.org/10.1007/s10531-010-9956-0

Kaartinen, R., & Roslin, T. (2011). Shrinking by numbers: Landscape context affects the species composition but not the quantitative structure of local food webs: Landscape effects on food webs. Journal of Animal Ecology, 80(3), 622–631. https://doi.org/10.1111/j.1365-2656.2011.01811.x

Kuzmanich, N., Giorgis, M. A., Bernaschini, L., Tavella, J., & Salvo, A. (2023). Galling insect communities mediate the effects of fire on their associated parasitoid communities. Insect Conservation and Diversity, 16(6), 817–827. https://doi.org/10.1111/icad.12674

Lampert, E. C., Dyer, L. A., & Bowers, M. D. (2010). Caterpillar Chemical Defense and Parasitoid Success: Cotesia congregata Parasitism of Ceratomia catalpae. Journal of Chemical Ecology, 36(9), 992–998. https://doi.org/10.1007/s10886-010-9840-0

Lev-Yadun, S. (2016). Gall Aposematism. In S. Lev-Yadun, Defensive (anti-herbivory) Coloration in Land Plants (pp. 213–223). Springer International Publishing. https://doi.org/10.1007/978-3-319-42096-7\_43

Lewis, O. T., Memmott, J., Lasalle, J., Lyal, C. H. C., Whitefoord, C., & Godfray, H. C. J. (2002). Structure of a diverse tropical forest insect–parasitoid community. Journal of Animal Ecology, 71(5), 855–873. https://doi.org/10.1046/j.1365-2656.2002.00651.x

López-Núñez, F. A., Ribeiro, S., Marchante, H., Heleno, R. H., & Marchante, E. (2019). Life inside a gall: Diversity, phenology and structure of Portuguese gall communities, their hosts, parasitoids and inquilines. Arthropod-Plant Interactions, 13(3), 477–488. https://doi.org/10.1007/s11829-018-9655-4

Loxdale, H. D., Lushai, G., & Harvey, J. A. (2011). The evolutionary improbability of ‘generalism’in nature, with special reference to insects. Biological Journal of the Linnean Society, 103(1), 1–18.

Luz, F. A., Goetz, A. P. M., & Mendonça, M. D. S. (2021). What drives gallers and parasitoids interacting on a host plant? A network approach revealing morphological coupling as the main factor. Ecological Entomology, 46(2), 334–341. https://doi.org/10.1111/een.12967

Maldonado-López, Y., Cuevas-Reyes, P., & Oyama, K. (2016). Diversity of gall wasps (Hymenoptera: Cynipidae) associated with oak trees (Fagaceae: Quercus) in a fragmented landscape in Mexico. Arthropod-Plant Interactions, 10(1), 29–39. https://doi.org/10.1007/s11829-015-9404-x

Maldonado-López, Y., de Araújo, W. S., González-Rodríguez, A., Oyama, K., Fagundes, M., Diaz-Castelazo, C., Sánchez-Echeverría, K., Borges, M., Pérez-López, G., & Cuevas-Reyes, P. (2022). Quantitative trophic networks of insect gallers and their parasitoids in the hybrid oak complex Quercus magnoliifolia x Quercus resinosa. Arthropod-Plant Interactions, 16(6), 631–643. https://doi.org/10.1007/s11829-022-09927-8

Maldonado-Lopez, Y., Prieto-Dueñas, I. S., Cuevas-Reyes, P., Vaca-Sánchez, M. S., Oyama, K., de Araújo, W. S., Zazá Borges, M. A., Fagundes, M., & Fernandes, G. W. (2023). Tri-Trophic Networks of Gall-Inducing Insects and Their Parasitoids Associated to Oak Species in Mexican Avocado Agrosystems: Importance of Native Forest Coverage and Local Climatic Variables (SSRN Scholarly Paper No. 4392343). https://doi.org/10.2139/ssrn.4392343

McAlpine, J. R. (1983). Climate of papua new guinea. Commonwealth scientific and industrial research organization in association …. https://openresearch-repository.anu.edu.au/handle/1885/115008

Memmott, J., Godfray, H. C. J., & Gauld, I. D. (1994). The structure of a tropical host-parasitoid community. Journal of Animal Ecology, 521–540.

Miller III, D. G., & Raman, A. (2019). Host–plant relations of gall-inducing insects. Annals of the Entomological Society of America, 112(1), 1–19.

Morris, R. J., Gripenberg, S., Lewis, O. T., & Roslin, T. (2014). Antagonistic interaction networks are structured independently of latitude and host guild. Ecology Letters, 17(3), 340–349. https://doi.org/10.1111/ele.12235

Morris, R. J., Lewis, O. T., & Godfray, H. C. J. (2004). Experimental evidence for apparent competition in a tropical forest food web. Nature, 428(6980), 310–313.

Morris, R. J., Lewis, O. T., & Godfray, H. C. J. (2005). Apparent competition and insect community structure: Towards a spatial perspective. Annales Zoologici Fennici, 449–462. https://www.jstor.org/stable/23735889

MU­Ller, C. B., Adriaanse, I. C. T., Belshaw, R., & Godfray, H. C. J. (1999). The structure of an aphid–parasitoid community. Journal of Animal Ecology, 68(2), 346–370. https://doi.org/10.1046/j.1365-2656.1999.00288.x

Nieves-Aldrey, J. L., & Butterill, P. T. (2014). First evidence of cynipids from the Oceanian Region: The description of Lithonecrus papuanus a new genus and species of cynipid inquiline from Papua New Guinea (Hymenoptera: Cynipidae, Synergini). Zootaxa, 3846(2), 221–234.

Nyman, T., Bokma, F., & Kopelke, J.-P. (2007). Reciprocal diversification in a complex plant-herbivore-parasitoid food web. BMC Biology, 5(1), 49. https://doi.org/10.1186/1741-7007-5-49

Paniagua, M. R., Medianero, E., & Lewis, O. T. (2009). Structure and vertical stratification of plant galler–parasitoid food webs in two tropical forests. Ecological Entomology, 34(3), 310–320. https://doi.org/10.1111/j.1365-2311.2008.01079.x

Pardikes, N. A., Lumpkin, W., Hurtado, P. J., & Dyer, L. A. (2018). Simulated tri-trophic networks reveal complex relationships between species diversity and interaction diversity. PLoS One, 13(3), e0193822.

Poisot, T., Bever, J. D., Nemri, A., Thrall, P. H., & Hochberg, M. E. (2011). A conceptual framework for the evolution of ecological specialisation. Ecology Letters, 14(9), 841–851. https://doi.org/10.1111/j.1461-0248.2011.01645.x

Price, P. W., Fernandes, G. W., & Waring, G. L. (1987). Adaptive nature of insect galls. Environmental Entomology, 16(1), 15–24.

Redfern, M., Shirley, P., & Bloxham, M. (2002). British plant galls: Identification of galls on plants and fungi. FSC publications. https://www.cabdirect.org/cabdirect/abstract/20033023178

Ripley, B., Venables, B., Bates, D. M., Hornik, K., Gebhardt, A., Firth, D., & Ripley, M. B. (2013). Package ‘mass.’ Cran r, 538, 113–120.

Rott, A. S., & Godfray, H. C. J. (2000). The structure of a leafminer-parasitoid community. Journal of Animal Ecology, 274–289.

Russo, R. (2006). Field guide to plant galls of California and other western states. University of California Press.

Schleuning, M., Fründ, J., Klein, A.-M., Abrahamczyk, S., Alarcón, R., Albrecht, M., Andersson, G. K., Bazarian, S., Böhning-Gaese, K., & Bommarco, R. (2012). Specialization of mutualistic interaction networks decreases toward tropical latitudes. Current Biology, 22(20), 1925–1931.

Schönrogge, K., Walker, P., & Crawley, M. J. (2000). Parasitoid and inquiline attack in the galls of four alien, cynipid gall wasps: Host switches and the effect on parasitoid sex ratios. Ecological Entomology, 25(2), 208–219. https://doi.org/10.1046/j.1365-2311.2000.00244.x

Stone, G. N., & Schönrogge, K. (2003). The adaptive significance of insect gall morphology. Trends in Ecology & Evolution, 18(10), 512–522.

Thompson, R. M., Brose, U., Dunne, J. A., Hall, R. O., Hladyz, S., Kitching, R. L., Martinez, N. D., Rantala, H., Romanuk, T. N., Stouffer, D. B., & Tylianakis, J. M. (2012). Food webs: Reconciling the structure and function of biodiversity. Trends in Ecology & Evolution, 27(12), 689–697. https://doi.org/10.1016/j.tree.2012.08.005

Tooker, J. F., Rohr, J. R., Abrahamson, W. G., & De Moraes, C. M. (2008). Gall insects can avoid and alter indirect plant defenses. New Phytologist, 178(3), 657–671. https://doi.org/10.1111/j.1469-8137.2008.02392.x

Tscharntke, T., Vidal, S., & Hawkins, B. A. (2001). Parasitoids of grass-feeding chalcid wasps: A comparison of German and British communities. Oecologia, 129(3), 445–451. https://doi.org/10.1007/s004420100733

Hartig, F. (2024). DHARMa: Residual diagnostics for hierarchical (multi-level/mixed) regression models. R package. https://CRAN.R-project.org/package=DHARMa

# **Tables**

Table 1. Categories of gall traits and taxon used as explanatory variables in GLMs. Size and similarity were used to derive apparency (Fig. 1); size was additionally evaluated in a sensitivity analysis replacing apparency (Table 2). "Plant module" denotes the architectural organ galled. Host gall abundance (minimum number of galled plant modules) was a covariate in all models. Counts per category in parentheses.

|  |  |  |
| --- | --- | --- |
| **Trait name** | **Description** | **Factor levels** |
| Apparency | Gall conspicuousness, based on size and similarity (see Fig. 1) | LO (33), MID (35), HI (10) |
| Size | Size of gall | SMALL (24), MID (38), LARGE (16) |
| Similarity | Similarity of galled tissue to regular plant tissue | LO (11), MID (36), HI (31) |
| Taxon | The gall-forming taxon. CECI = Cecidomyiidae, HEMI = Hemiptera, OTHE = Others, UNKN = Unknown. Others includes Thysanoptera, Lepidoptera, Hymenoptera and Agromyzidae (Diptera) | CECI (32), HEMI (15), OTHE (12), UNKN (19) |
| Plant module | The type of plant module galled: LEAF = leaves; OTHER = non-leaf modules; COMP = complex: multiple module types galled | LEAF (55), OTHER (17), COMP (6) |
| Pubescence | YES/NO, gall surface covered in hairs or not | HAIRY (12), NOT HAIRY (66) |
| Openness | Whether the gall had some form of opening (TRUE) or was fully enclosed (FALSE) | TRUE (21), FALSE (57) |

Table 2. Negative binomial GLM comparison for parasitoid species richness (response = number of parasitoid species per gall morphospecies). All models include log(host gall abundance) unless noted. AIC = Akaike information criterion; e.d. = explained deviance (%). Coefficient tables: Table S3; Cecidomyiidae-only models: Table S4.

|  |  |  |
| --- | --- | --- |
| Model | AIC | e.d. (%) |
| Abundance only | 261.4 | 29.2 |
| Apparency + abundance | 250.7 | 42.6 |
| Size (categorical) + abundance | 257.2 | 36.9 |
| Size (ordinal) + abundance | 260.3 | 32.2 |
| Taxon + abundance | 251.3 | 43.5 |
| Openness + abundance | 257.6 | 34.6 |
| Plant module + abundance | 263.9 | 30.6 |
| Surface + abundance | 263.2 | 29.5 |
| Full model (with apparency) | 251.9 | 52.3 |
| Final: apparency + taxon + abundance | 246.8 | 50.2 |
| Full model (with size) | 250.7 | 53.0 |
| Final size sensitivity: size + taxon + abundance | 248.5 | 48.8 |
| Cecidomyiidae-only: apparency + plant module\* | 138.2 | 47.4 |

\*Without log(abundance); see Table S4.

**Figures**


Figure 1. Gall morphotypes classified by gall apparency (cell colour), based on categorisation of similarity (columns) and size (rows). Similarity categories reflect how visually similar each gall is to the plant organ on which it occurs (high, mid, low). Size classes are defined as small (< 5 mm), mid (5–15 mm), and large (> 15 mm). One example morphotype is provided per combination of similarity and size. Numbers in each row and column denote the total number of gall morphotypes in that category.


Figure 2. Quantitative tripartite network for a community of host plant species (lower level), galling insect species (middle levels), and parasitoid species (top level). Node (species) widths and interaction segments are proportional to the relative abundance per trophic level, i.e. uniform sampling of host plants, the minimum abundance of galled plant modules (see Methods for details), and parasitoid abundances, respectively. Node width scales are shown for herbivores and parasitoids. For host plants and herbivores, nodes are filled according to the log abundance of their herbivores and parasitoids, respectively. The network is ordered by the host plant phylogeny (see supplementary), from gymnosperms to basal Eudicots, Rosids I and II and Asterids (left to right). The remaining trophic levels were ordered to minimise the number of interaction crossings.


Figure 3. Accumulation curves showing interpolated (solid lines) and extrapolated (dashed lines) estimates for a) parasitoid species richness and b) sample coverage (the proportion of the total individuals in the community that belong to species already detected in the sample) for all three study sites combined


Figure 4. Parasitoid overlap graph for 42 gall-forming host species, based on the potential for apparent competition (PAC) between hosts with shared parasitoids. The proportion of turquoise fill for each species node represents the extent to which that species is responsible for the supply of its own parasitoids. Connecting line segments represent shared enemies between pairs of hosts nights and the width of the line at each species represents the importance of the other species as a source of its parasitoids . Species with no connecting segments have no shared parasitoids.


Figure 5. The relationship between the number of parasitoid species and (minimum) host gall abundance for a) gall apparency and b) gall-forming taxon. Curves were fitted as predicted by respective generalised linear models of both explanatory variables, in which they were significant components (P < 0.001; Table S4).
